# Mitigating workplace bullying through authentic leadership: Evidence from hospital nurses in Jordan

**DOI:** 10.1371/journal.pone.0359313

**Published:** 2026-10-21

**Authors:** Rahaf Abdulsalam Al-Laham, Ali Moh’d Saleh, Waddah Mohammad D’emeh, Muhammad W. Darawad, Duaa Izzat Zaidiyah, Tariq Noureddin Mkhaimer

**Affiliations:** 1 Faculty of Nursing, Ibn Sina University for Medical Sciences, Amman, Jordan; 2 Department of Community Health Nursing, School of Nursing, The University of Jordan, Amman, Jordan; 3 Department of Clinical Nursing, School of Nursing, The University of Jordan, Amman, Jordan; 4 Dr. Sulaiman Al Habib Medical Group, Riyadh, Saudi Arabia; Mutah University, JORDAN

## Abstract

**Background:**

Workplace bullying constitutes a major challenge within healthcare settings, particularly among nursing professionals. High prevalence rates of workplace bullying among nurses have been documented globally, underscoring the urgent need for effective leadership strategies to prevent, address, and mitigate bullying behaviors within healthcare environments.

**Aim:**

To examine the relationship between authentic leadership behaviors and the occurrence of workplace bullying among registered nurses in Jordan.

**Methods:**

A correlational cross-sectional design was employed in this study. A convenience sample of 205 registered nurses was recruited from two hospitals in Jordan, comprising one university-affiliated hospital and one private hospital. Data were collected from March 1 to June 1, 2023 using a self-administered questionnaire comprising the Authentic Leadership Questionnaire and the Negative Acts Questionnaire–Revised. Descriptive and inferential statistical analyses were performed to analyze the data.

**Results:**

The mean score for supervisors’ authentic leadership as perceived by nurses was 2.78 (SD = 0.75), whereas the mean score for overall workplace bullying was 2.17 (SD = 0.94). The prevalence of workplace bullying among participants was 64.9% (N = 133), comprising 11.7% (N = 24) who reported occasional bullying and 53.2% (N = 109) who reported severe bullying. The findings demonstrated a moderate negative correlation between authentic leadership and workplace bullying (*r* = −0.51, p < 0.001). Furthermore, the multiple regression model was statistically significant (*F* (17, 187) = 5.94, *p* < 0.001, adjusted *R*^*2*^ = 0.292) indicating that hospital type, working unit, and the transparency dimension of authentic leadership were significant predictors of workplace bullying.

**Conclusion:**

The study highlights an association between authentic leadership and lower perceptions of workplace bullying in healthcare settings. The results suggest that strengthening authentic leadership development initiatives may be associated with reductions in workplace bullying. The multifactorial nature of bullying underscores the need for multilevel organizational strategies to create safer and more supportive nursing environments.

## Introduction

The healthcare system faces multiple complex challenges, including a shortage of nurses, workplace bullying, an increasing incidence of infectious and emerging diseases, a growing aging population, financial constraints, and a highly demanding work environment [1]. Workplace bullying constitutes a significant challenge in healthcare settings, particularly among nursing staff, where high-stress environments may exacerbate interpersonal conflict and undermine team dynamics. Workplace bullying (WPB) is defined as repeated and persistent negative behavior, enacted by an individual or group with perceived or actual power, directed toward another individual who experiences difficulty defending against such behavior over a prolonged period (e.g., at least six months) [2].

According to the World Health Organization (WHO), the global nursing shortage is projected to reach approximately 4.1 million by 2030 [3]. A toxic work environment, including workplace bullying, has been identified as a contributing factor to nurses’ intention to leave the profession [4]. The prevalence of workplace bullying varies across industries worldwide, with certain occupations facing a higher risk of exposure than others [5]. In healthcare settings, both the prevalence and characteristics of workplace bullying differ across and within countries [6], with reported prevalence rates of 47.1% in Asia, 36.1% in Australia, 18.4% in Europe, and 24.5% in North America [7]. Nurses, in particular, are considered especially vulnerable to workplace bullying, with reported prevalence rates ranging from 25.6% to 73.1% [8]. In Jordan, one study reported that 43% of nurses experienced severe workplace bullying, while more than 31% indicated that they occasionally encountered bullying on the job [9]. Similarly, another study found a high prevalence of workplace bullying among nurses, with a reported rate of 53.4% [4]. These consistently high rates underscore the urgent need for nurse leaders to address and mitigate workplace bullying within healthcare settings.

Workplace bullying has been consistently associated with adverse consequences at multiple levels, extending beyond individual employee well-being to affect work performance, patient care, and organizational functioning. At the individual level, evidence indicates that exposure to bullying is associated with poorer psychosocial well-being, including psychological stress, depression, burnout, and reduced self-worth [10–12], as well as physical health problems such as sleep disturbances, headaches, and gastrointestinal symptoms [7,13]. These effects may subsequently impair employees’ ability to function effectively at work, contributing to reduced concentration, disrupted professional relationships, and compromised work performance [8,14]. Importantly, the consequences may extend beyond the targeted employee: studies have linked workplace bullying with adverse patient outcomes, including treatment errors, patient falls, and patient complaints [15]. At the organizational level, bullying is further associated with lower job satisfaction, increased intention to leave, staff turnover, and additional organizational costs [14]. Taken together, these findings suggest that workplace bullying is not merely an interpersonal problem but a multidimensional occupational stressor with potentially cascading consequences for employee health, workplace functioning, patient safety, and organizational sustainability.

Addressing workplace bullying within healthcare systems requires organizational conditions that promote respectful, ethical, and supportive interactions, with leadership representing a potentially important determinant of such conditions. Effective leadership can contribute to staff and patient satisfaction and facilitate the achievement of organizational goals [16]. Rather than viewing leadership solely as the exercise of authority, contemporary perspectives conceptualize it as a relational process through which leaders and followers interact to coordinate collective actions toward shared goals [17]. Within this broader perspective, authentic leadership has emerged as a particularly relevant leadership approach due to its emphasis on self-awareness, relational transparency, ethical decision-making, and the development of positive psychological capacities, including confidence, hope, optimism, and resilience [18]. These characteristics may be particularly salient in healthcare settings, where complex interpersonal relationships, high job demands, and emotionally challenging working conditions can contribute to workplace conflict and bullying.

The existing evidence suggests that leadership may influence workplace bullying through both direct interpersonal behaviors and broader organizational processes. Leadership characterized by unfairness, lack of support, and poor relationship-building has been associated with greater exposure to workplace bullying [19], while conflict between leaders and employees and unfavorable leadership styles have also been identified as important organizational correlates [20]. Conversely, relationally oriented approaches, particularly authentic leadership, have been associated with lower levels of workplace bullying [19,21–24]. Taken together, these results suggest that the relationship between leadership and workplace bullying is not simply a matter of individual leader behavior; rather, leadership may shape the relational climate, psychological safety, and approaches to conflict within the organization.

Accordingly, authentic leadership may be associated with reducing workplace bullying by fostering ethical and transparent relationships, self-awareness, and constructive problem solving, which can create conditions less conducive to bullying and more supportive of positive workplace interactions [25]. Furthermore, Avolio et al. [18] emphasized the pivotal role of emotions in enabling individuals to develop adaptive responses to workplace setbacks and stressors. By recognizing and constructively leveraging the information embedded in emotional experiences, authentic leaders can shape followers’ cognitive processes and behavioral responses, thereby strengthening their capacity to cope with interpersonal difficulties and effectively navigate complex organizational challenges.

The present study addresses a critical gap in the literature by examining the influence of authentic leadership on bullying behaviors within Jordanian hospitals. Authentic leadership characterized by self-awareness, relational transparency, balanced processing of information, and an internalized moral perspective [26], is conceptualized as a potential mitigating factor against workplace bullying and its negative outcomes. Although authentic leadership has been increasingly examined in nursing research, the existing literature from Jordan has predominantly focused on outcomes such as mental health, job satisfaction, work engagement, and turnover [27–30]. In contrast, substantially less is known about whether and to what extent authentic leadership is associated with workplace bullying, particularly among Jordanian nurses across different hospital sectors. This represents an important gap because the organizational and leadership contexts in which nurses work may influence both the experience of workplace bullying and the potential protective role of authentic leadership. Therefore, examining the relationship between authentic leadership and workplace bullying among Jordanian nurses across different hospital sectors may provide context-specific evidence that extends the existing literature and informs leadership strategies aimed at creating healthier and safer healthcare work environments.

The significance of this study lies in its examination of the potential role of authentic leadership in addressing workplace bullying among hospital nurses within the Jordanian healthcare context. Hospital nurses constitute a particularly important population because they work in complex and demanding environments characterized by continuous patient care, multidisciplinary collaboration, hierarchical relationships, high workloads, and frequent interpersonal interactions. These conditions may increase the risk of workplace conflict and bullying while simultaneously underscoring the importance of supportive, transparent, and ethical leadership. Examining this relationship in Jordan is particularly important because evidence generated in other healthcare systems may not be directly transferable to the Jordanian context. Leadership practices, organizational structures, professional relationships, and workplace norms can vary across healthcare systems and cultural settings, potentially shaping both the prevalence of workplace bullying and nurses’ perceptions and experiences of leadership behaviors. Generating context-specific evidence is therefore essential for informing evidence-based policies, leadership development and training, and organizational interventions designed to foster healthier work environments and, ultimately, enhance the quality and safety of patient care within the Jordanian healthcare system.

The aim of the current study is to examine the relationship between authentic leadership behaviors and the occurrence of workplace bullying among registered nurses in Jordan. To achieve this aim, the study addresses the following research questions: (1) What are the levels of authentic leadership, as perceived by nurses in Jordanian hospitals? (2) What are the levels of workplace bullying—specifically work-related, person-related, and physically intimidating bullying—among Jordanian nurses? (3) Is there a significant relationship between authentic leadership and workplace bullying in Jordanian hospitals? (4) To what extent do authentic leadership and nurses’ demographic characteristics predict workplace bullying?

## Materials and methods

### Design

A correlational cross-sectional design was utilized to investigate the relationship between authentic leadership behaviors and the occurrence of workplace bullying among registered nurses in Jordan.

### Setting and sampling

The current study was conducted in two hospitals in Amman, the capital city of Jordan: one university-affiliated hospital and one private hospital. These institutions were selected because they are among the largest in their respective sectors. Both hospitals are accredited by the Joint Commission International and the Health Care Accreditation Council. A convenience sample of registered nurses was recruited from each selected hospital. Nurses who met the following inclusion criteria were invited to participate: (1) at least six months of work experience at the same hospital and (2) a bachelor’s degree in nursing. Nurse supervisors and head nurses were excluded from the study.

The sample size was calculated using G*Power (version 3.1). For multiple regression analysis, the required sample size was determined based on a significance level of α = 0.05, an estimate of power(1-β) of 0.95, and a medium effect size of f² = 0.15. The estimated minimum sample size was 178 participants. The final sample size in the current study was 205 participants. The sample was recruited from both participating hospitals. Of the 641 eligible nurses at the university-affiliated hospital, 106 nurses were recruited, while 99 nurses were recruited from the private hospital, where 283 nurses were eligible to participate.

### Measures

Data were collected using a self-administered questionnaire comprising three sections. The first section included a demographic data sheet. The second section consisted of the Authentic Leadership Questionnaire. The third section included the Negative Acts Questionnaire-Revised. All measures were administered in English, as nursing education in Jordan is conducted in English.

A demographic data sheet was used to collect participants’ characteristics. It included eight variables: age, gender, education level, marital status, years of professional experience, years of experience in the current unit, type of hospital, and unit type.

Authentic Leadership Questionnaire (ALQ) [26] was used to assess authentic leadership. The questionnaire consists of 16 items across four subscales: self-awareness (4 items), transparency (5 items), moral/ethical (4 items), and balanced processing (3 items). Responses are rated on a 5-point Likert scale ranging from 0 (not at all) to 4 (frequently, if not always). Participants were asked to indicate the extent to which each statement reflected the leadership style of their immediate supervisors. Subscale scores and an overall authentic leadership score were calculated by averaging the relevant items, with higher scores indicating greater demonstration of authentic leadership behaviors. The ALQ has demonstrated good validity and reliability. Reported Cronbach’s alpha coefficients for the total scale and its subscales range from.76 to.92, indicating acceptable to excellent internal consistency [26]. In the current study, the ALQ demonstrated excellent internal consistency, with a Cronbach’s alpha of.95 for the overall scale. The alpha coefficients for the subscales were.89 for self-awareness,.87 for transparency,.83 for moral/ethical perspective, and.82 for balanced processing, indicating good internal consistency across all subscales.

The Negative Acts Questionnaire-Revised (NAQ-R) [31] was used to measure workplace bullying. The NAQ-R was designed to assess perceived exposure to bullying and victimization in various occupational settings. The scale consists of 22 items encompassing three dimensions: work-related (7 items), person-related (12 items), and physically intimidating bullying (3 items). Participants were asked to respond using a five-point Likert scale to represent the perceived frequency of exposure to negative acts ranging from 1 (never) to 5 (daily). Subscale scores as well as an overall workplace bullying score were calculated by averaging the relevant items, with higher scores indicating higher degrees of bullying. The total score range from 22 to 110. Based on established cut-off values, participants scoring <33 were classified as “not bullied,” those scoring between 33 and 45 as “occasionally bullied,” and those scoring >45 as “severely bullied” [32]. The NAQ-R scales’ psychometric qualities have been studied with reported Cronbach’s alpha of 0.90 [31], indicating high internal consistency. In the current study, the NAQ-R demonstrated excellent internal consistency, with a Cronbach’s alpha of.96 for the overall scale. The Cronbach’s alpha coefficients for the work-related, person-related, and physically intimidating bullying subscales were.89,.94, and.72, respectively.

### Ethical considerations

Ethical approval was obtained from the Institutional Review Board (IRB) of the participating hospitals prior to data collection (No. 10/2023/5895, No. 5/1/112976). All participants were provided with a written informed consent form accompanied by a cover letter outlining the study’s purpose, significance, procedures, and participants’ rights. Participants were informed that their participation was entirely voluntary and that they could withdraw from the study at any time without penalty. Confidentiality and anonymity were strictly maintained; no personally identifiable information was collected. All questionnaires and study materials were securely stored in a locked cabinet in the principal investigator’s office. Additionally, permission to use the study instruments was obtained from the original developers. Additional information regarding the ethical, cultural, and scientific considerations specific to inclusivity in global research is included in the Supporting Information (S1 Checklist).

### Data collection procedures

Following ethical approval from the participating hospitals, one of the researchers met with the Chief Nursing Officer (CNO) at each site to explain the study objectives, data collection procedures, estimated time required to complete the questionnaire, the target number of registered nurses to be recruited from each hospital, and to request logistical support. Subsequently, nurse managers from selected departments were consulted individually to determine the most appropriate strategy for approaching eligible registered nurses without disrupting routine clinical operations.

A convenience sampling approach was employed to recruit registered nurses based on their availability during the data collection period. All eligible nurses present during this period were invited to participate. One of the researchers was present in the participating hospitals three days per week, covering different shifts, to facilitate recruitment. Nurses who agreed to participate were asked to complete the questionnaire, seal it in the provided envelope, and deposit it in secure collection boxes located in the nurse managers’ offices. Participants were also informed that they could contact the research team using the details provided in the consent form if they had any questions. Completed questionnaires were collected regularly until the required sample size was achieved. Data were collected from March 1 to June 1, 2023 at the private hospital and from March 7 to June 1, 2023 at the university-affiliated hospital.

### Data analysis

Data were analyzed using IBM SPSS Statistics (Version 26). Prior to analysis, the dataset was entered, screened, and checked for accuracy and completeness. Descriptive statistics, including means, standard deviations, ranges, frequencies, and percentages, were computed to summarize sample characteristics, authentic leadership and its subscales, and workplace bullying and its subscales. Pearson’s correlation coefficient (*r*) was used to examine the relationships between authentic leadership and workplace bullying. In addition, standard multiple regression analysis was conducted to determine the extent to which demographic variables (age, years of professional experience, sex, educational level, marital status, and type of hospital) and the dimensions of authentic leadership (transparency, moral/ethical perspective, balanced processing, and self-awareness) predict workplace bullying.

Prior to conducting the inferential analyses, the assumptions underlying the statistical procedures were assessed. The distributions of continuous variables were examined using histograms and Q–Q plots, and linearity was assessed using scatterplots. No substantial departures from normality or linearity were observed, and no influential outliers were identified. As these assumptions were adequately satisfied, Pearson’s correlation was used to examine the strength and direction of linear associations between the study variables. For the multiple regression analysis, the normality and homoscedasticity of residuals were assessed using Q–Q plots and residual-versus-fitted-value plots, respectively. Multicollinearity was examined using tolerance and variance inflation factor (VIF) values, with all VIF values below 5 and tolerance values above 0.20 indicating no problematic multicollinearity. A two-tailed *p* value < 0.05 was considered statistically significant.

## Results

### Sample characteristics

A total of 230 nurses were approached, and all were assessed as eligible and invited to participate in the study using convenience sampling. Of these, 205 returned the questionnaires, yielding a response rate of 89.1%, while 25 questionnaires were not returned. The reasons for non-return were unknown. All 205 returned questionnaires were complete, met the inclusion criteria, and were therefore included in the final analysis. As shown in Table 1, participants’ ages ranged from 22 to 56 years (M = 31.5). The majority of participants were female (62.9%, n = 129). In terms of educational level, most participants (89.8%, n = 184) held a bachelor’s degree in nursing, whereas 10.2% (n = 21) held a master’s or doctoral degree. Regarding marital status, over half of the participants were married (57.6%, n = 118), followed by single individuals (39.0%, n = 80), while a small proportion were divorced (3.4%, n = 7). Professional experience ranged from 1 to 34 years, with a mean of 9.0 years (SD = 5.84). More than half of the participants were employed in university-affiliated hospitals (51.7%, *n* = 106), while 30.2% (n = 62) worked in medical–surgical units.

**Table 1:** Descriptive statistics of participants’ demographic characteristics (N = 205).

Variable	Frequency (%)	M (SD)	Range
Age in years		31.5 (6.24)	22-56
Years of professional experience		9.0 (5.84)	1-34
Sex			
Male	76 (37.1)		
Female	129 (62.9)		
Level of education			
Bachelor	184 (89.8)		
Postgraduate	21 (10.2)		
Marital status			
Single	80 (39.0)		
Married	118 (57.6)		
Divorced	7 (3.4)		
Type of hospital			
University affiliated	106 (51.7)		
Private	99 (48.3)		
Type of unit			
Medical-surgical	62 (30.2)		
ER	17 (8.3)		
Critical care units	44 (21.5)		
Pediatric	21 (10.2)		
OR	20 (9.8)		
Obstetrics and Gynecology	20 (9.8)		
Others	21 (10.2)		

M: Mean, SD: Standard deviation, %: Percentage, ER: Emergency Unit, OR: Operation Room.

### The perception of authentic leadership

To assess the level of supervisors’ authentic leadership as perceived by nurses, descriptive statistics were computed. Means and standard deviations were calculated for the overall authentic leadership score and its subscales: transparency, moral/ethical perspective, balanced processing, and self-awareness. As presented in Table 2, the overall mean score for authentic leadership was 2.78 (SD = 0.75). The subscale mean scores were closely comparable, ranging from 2.76 to 2.80, indicating minimal variation across the four dimensions of authentic leadership. Balanced processing (M = 2.80, SD = 0.83) and self-awareness (M = 2.80, SD = 0.84) had marginally higher mean scores, whereas transparency (M = 2.76, SD = 0.84) and moral/ethical perspective (M = 2.76, SD = 0.84) had marginally lower mean scores. Overall, the differences between the subscale means were small and should not be interpreted as practically meaningful.

**Table 2:** Descriptive statistics of authentic leadership perceptions among nurses (N = 205).

Variable	M (SD)
Authentic leadership*	**2.78 (0.75)**
Transparency	2.76 (0.84)
Moral/ethical	2.76 (0.80)
Balanced processing	2.80 (0.83)
Self-awareness	2.80 (0.84)

* Scale composite score.

M: Mean, SD: Standard deviation.

### The perception of workplace bullying

Descriptive statistics were used to assess nurses’ perceptions of workplace bullying. Means and standard deviations were calculated for overall workplace bullying and its subscales: work-related bullying, person-related bullying, and physically intimidating bullying. As shown in Table 3, the mean score for overall workplace bullying was 2.17 (SD = 0.94). Across the subscales, mean scores ranged from 1.98 to 2.28. The highest mean score was observed for work-related bullying (M = 2.28, SD = 0.99), followed by person-related bullying (M = 2.16, SD = 0.98). In contrast, physically intimidating bullying had the lowest mean score (M = 1.98, SD = 0.97). Moreover, the prevalence of workplace bullying was determined using established cut-off values [32], as presented in Table 4. The results indicated that 35.1% (n = 72) of participants were classified as not bullied, 11.7% (n = 24) as occasionally bullied, and 53.2% (n = 109) as severely bullied.

**Table 3:** Descriptive statistics of workplace bullying perceptions among nurses (N = 205).

Variable	M (SD)
Workplace bullying*	**2.17 (0.94)**
**Work related bullying**	**2.28 (0.99)**
1- Someone withholding information which affects your performance	2.25 (1.29)
3- Being ordered to do work below your level of competence	2.18 (1.21)
14- Having your opinions ignored	2.18 (1.24)
16- Being given tasks with unreasonable deadlines	2.14 (1.20)
18- Excessive monitoring of your work	2.37 (1.29)
19- Pressure not to claim something to which by right you are entitled (e.g.,sick leave, holiday)	2.38 (1.28)
21- Being exposed to an unmanageable workload	2.45 (1.35)
**Person related bullying**	**2.16 (0.98)**
2- Being humiliated or ridiculed in connection with your work	1.92 (1.19)
4- Having key areas of responsibility removed or replaced with more trivial or unpleasant tasks	2.13 (1.16)
5- Spreading of gossip and rumors about you	2.18 (1.31)
6- Being ignored or excluded	2.14 (1.25)
7- Having insulting or offensive remarks made about your person, your attitudes, or your private life	2.24 (1.31)
10- Hints or signals from others that you should quit your job	2.24 (1.26)
11- Repeated reminders of your errors or mistakes	2.35 (1.32)
12- Being ignored or facing a hostile reaction when you approach	2.20 (1.15)
13- Persistent criticism of your errors or mistakes	2.15 (1.17)
15- Practical jokes carried out by people you don’t get along with	2.16 (1.20)
17- Having allegations made against you	2.06 (1.13)
20- Being the subject of excessive teasing and sarcasm	2.14 (1.24)
**Physically intimidating bullying**	**1.98 (0.97)**
8- Being shouted at or being the target of spontaneous anger	2.25 (1.35)
9- Intimidating behaviors such as finger-pointing, invasion of personal space, shoving, blocking your way	2.14 (1.24)
22- Threats of violence or physical abuse or actual abuse	1.55 (0.98)

* Scale composite score.

M: Mean, SD: Standard deviation.

**Table 4:** The prevalence of workplace bullying among nurses (N = 205).

Category	Frequency	Percentage
Not bullied	72	35.1%
Occasionally bullied	24	11.7%
Severely bullied	109	53.2%

Furthermore, individual items within each subscale were examined as shown in Table 3. The analysis indicated that mean scores for workplace bullying items ranged from 1.55 to 2.45. Within the work-related bullying subscale, the highest mean score was observed for the item “Being exposed to an unmanageable workload” (M = 2.45, SD = 1.35), whereas the lowest mean score was for “Being given tasks with unreasonable deadlines” (M = 2.14, SD = 1.20). Regarding person-related bullying, the highest mean score was noted for “Repeated reminders of your errors or mistakes” (M = 2.35, SD = 1.32), while the lowest mean score was for “Being humiliated or ridiculed in connection with your work” (M = 1.92, SD = 1.19). In terms of physically intimidating bullying, the highest mean score was found for “Being shouted at or being the target of spontaneous anger” (M = 2.25, SD = 1.35), whereas the lowest mean score was observed for “Threats of violence or physical abuse or actual abuse” (M = 1.55, SD = 0.98).

### The relationship between authentic leadership and workplace bullying

To examine the relationship between authentic leadership and workplace bullying, Pearson’s correlation coefficient (*r*) was calculated. As presented in Table 5, the analysis revealed moderate negative correlations between authentic leadership (including its subscales) and workplace bullying (including its subscales). Overall authentic leadership was moderately and negatively correlated with overall workplace bullying (*r* = −0.51, p < 0.001), work-related bullying (*r* = −0.48, p < 0.001), person-related bullying (*r* = −0.51, p < 0.001), and physically intimidating bullying (*r* = −0.45, p < 0.001).

**Table 5:** Correlations between authentic leadership and workplace bullying and their subscales (N = 205).

Variables	Overall workplace bullying	Work related	Person related	Physically intimidating
Overall authentic leadership	−0.51**	−0.48**	−0.51**	−0.45**
Transparency	−0.53**	−0.48**	−0.53**	−0.47**
Moral/ethical	−0.43**	−0.41**	−0.41**	−0.37**
Balanced processing	−0.45**	−0.41**	−0.46**	−0.41**
Self awareness	−0.43**	−0.43**	−0.42**	−0.36**

**: Correlation is significant at alpha < 0.01 (2-tailed).

Regarding the transparency subscale, significant moderate and negative correlations were observed with overall workplace bullying (*r* = −0.53, p < 0.001), work-related bullying (*r* = −0.48, p < 0.001), person-related bullying (*r* = −0.53, p < 0.001), and physically intimidating bullying (*r* = −0.47, p < 0.001). Similarly, the moral/ethical subscale was moderately and negatively correlated with overall workplace bullying (*r* = −0.43, p < 0.001), work-related bullying (*r* = −0.41, p < 0.001), person-related bullying (*r* = −0.41, p < 0.001), and physically intimidating bullying (*r* = −0.37, p < 0.001). In terms of balanced processing, moderate and negative correlations were found with overall workplace bullying (*r* = −0.45, p < 0.001), work-related bullying (*r* = −0.41, p < 0.001), person-related bullying (*r* = −0.46, p < 0.001), and physically intimidating bullying (*r* = −0.41, p < 0.001). Finally, the self-awareness subscale was also moderately and negatively correlated with overall workplace bullying (*r* = −0.43, p < 0.001), work-related bullying (*r* = −0.43, p < 0.001), person-related bullying (*r* = −0.42, p < 0.001), and physically intimidating bullying (*r* = −0.36, p < 0.001).

### Predictors of workplace bullying

To examine the extent to which age, professional experience, sex, level of education, marital status, type of hospital, and the dimensions of authentic leadership (transparency, moral/ethical perspective, balanced processing, and self-awareness) predict workplace bullying, a standard multiple regression analysis was conducted. The overall model was statistically significant, *F* (17, 187) = 5.94, *p* < 0.001, explaining 29% of the variance in workplace bullying (adjusted *R*^*2*^ = .29). Among the variables entered into the model, type of hospital, working unit, and the transparency dimension of authentic leadership emerged as significant predictors. Specifically, nurses employed in private hospitals were less likely to perceive workplace bullying compared to those working in university-affiliated hospitals (*β* = −0.15, *t* = −2.25, *p* = 0.026). In addi*t*ion, nurses working in emergency rooms were more likely to perceive workplace bullying than those in medical–surgical units (*β* = 0.16, *t* = 2.31, *p* = 0.022). Fur*t*hermore, higher perceived levels of supervisor transparency were associated with lower levels of workplace bullying (*β* = −0.38, *t* = −3.71, *p* < 0.001). In con*t*rast, age, professional experience, sex, level of education, marital status, and the remaining authentic leadership dimensions (moral/ethical perspective, balanced processing, and self-awareness) were not significant predictors of workplace bullying (see Table 6).

**Table 6:** Standard multiple regression predicting workplace bullying as perceived by nurses (N = 205).

Predictor	B	SE	β	t	*p*	95% CI for B	
						Lower	Upper
Constant	3.958	0.471		8.400	< 0.001	3.028	4.887
Age	0.005	0.015	0.036	0.357	0.721	−0.025	0.035
Professional experience	−0.020	0.016	−0.126	−1.289	0.199	−0.051	0.011
Sex							
Male	0.068	0.130	0.035	0.524	0.601	−0.188	0.324
Female (Ref.)							
Level of education							
Postgraduate	0.064	0.195	0.021	0.331	0.741	−0.320	0.449
Bachelor (Ref.)							
Marital status							
Single	0.045	0.134	0.023	0.332	0.740	−0.221	0.310
Divorced	0.277	0.316	0.054	0.878	0.381	−0.346	0.899
Married (Ref.)							
Type of hospital							
Private	−0.286	0.127	−0.152	−2.247	0.026	−0.537	−0.035
University-affiliated (Ref.)							
Working unit							
ER	0.528	0.229	0.155	2.308	0.022	0.077	0.980
Critical care units	−0.199	0.164	−0.087	−1.209	0.228	−0.523	0.125
Pediatric	0.148	0.213	0.048	0.694	0.489	−0.272	0.568
OR	−0.086	0.215	−0.027	−0.401	0.689	−0.510	0.338
Obstetrics and Gynecology	−0.094	0.216	−0.030	−0.437	0.663	−0.520	0.331
Others	−0.020	0.214	−0.006	−0.092	0.927	−0.441	0.402
Medical-surgical (Ref.)							
Transparency	−0.424	0.114	−0.378	−3.711	< 0.001	−0.649	−0.198
Moral/ethical	0.074	0.134	0.063	0.548	0.584	−0.191	0.338
Balanced processing	−0.188	0.128	−0.165	−1.459	0.146	−0.441	0.066
Self awareness	−0.065	0.140	−0.058	−0.467	0.641	−0.341	0.210

Ref.: reference category, B: unstandardized beta, SE: standard error for the unstandardized beta β: standardized beta, CI: confidence interval, *p*: level of significance at *p* < 0.05.

## Discussion

The findings suggest that nurses perceived their managers as demonstrating a moderate level of authentic leadership, with comparable levels across the four dimensions. These findings are consistent with prior research conducted in Jordan [28], Canada [33], and Oman [34], suggesting a relatively stable pattern of authentic leadership perceptions across diverse healthcare contexts. However, the levels reported in the present study were higher than those identified in other regional and international studies, including those conducted in Jordan [22,29,30], Saudi Arabia [35], and South Korea [36]. This discrepancy may reflect contextual and organizational differences, including evolving leadership expectations and professional standards within Jordanian healthcare settings.

The study showed a growing emphasis on authentic leadership as a desirable managerial approach among nurse managers. Such trends could be associated with ongoing reforms in healthcare systems, increased investment in leadership development, and stricter qualification requirements for nurse managers, particularly in private and university-affiliated hospitals. For instance, the requirement of advanced academic credentials (e.g., master’s degrees or specialized diplomas in nursing management) may contribute to enhanced leadership competencies. Additionally, national efforts to strengthen leadership capacity through continuing education programs, structured training, and professional development initiatives may further explain the relatively higher levels observed in this study.

In the present study, the balanced processing and self-awareness subscales yielded the highest mean scores among all dimensions of authentic leadership. These results are consistent with prior research. For instance, Almudhaybiri et al. [35] reported that balanced processing had the highest mean score among authentic leadership subscales among Saudi nurses, whereas other studies from Jordan, Oman, and China have identified self-awareness as the highest-scoring dimension [28,34,37]. However, the present results contrast with studies reporting higher mean scores for transparency [36] and moral/ethical perspectives [22,29,30].

Despite the relatively small variation between the lowest and highest mean scores across subscales (ranging from 2.76 to 2.80), nurses in this study perceived their managers as placing greater emphasis on soliciting diverse viewpoints before making critical decisions and demonstrating awareness of their own strengths, weaknesses, and the impact of their actions on others. Conversely, lower emphasis was perceived in the domains of transparency and moral/ethical leadership. This may be attributed to the challenges managers face in fostering open communication and consistently addressing complex ethical issues in practice. Overall, these results highlight specific dimensions of authentic leadership that warrant further attention among Jordanian nurse managers.

The analysis indicated a high prevalence of workplace bullying among nurses, with approximately 65% reporting exposure to bullying at occasional or severe levels. This prevalence aligns with previous studies conducted in Jordan, which reported rates ranging from 51.2% to 75.8% [4,9,22,38]. Regarding the level of workplace bullying, the mean score observed in the current study is consistent with those reported in prior research conducted in the same context [4,9,27,38]. In comparison with international studies, the level of workplace bullying reported in this study appears higher than levels documented in studies from Canada [23], China [39], South Korea [19], and Taiwan [40], but lower than those reported in Egypt [21].

The high prevalence and elevated levels of workplace bullying identified in the present study may be attributed to multiple systemic factors within the healthcare environment. These include high job demands and role conflict among nurses [41], the absence of well-structured organizational systems, unclear job descriptions and scopes of practice, nursing shortages, ineffective teamwork, and limited professional autonomy [9]. Furthermore, Karatuna et al. [13] suggested that the high prevalence of workplace bullying in Middle Eastern countries may be partly explained by the lack of effectively implemented antibullying policies.

Among the dimensions of workplace bullying, work-related bullying showed the highest level, whereas physically intimidating bullying showed the lowest. These findings align with previous research conducted in various countries [4,9,21,23,27,38]. The results suggest that nurses perceived work-related bullying as the primary source of bullying, encompassing behaviors that target job performance, professional competence, and work assignments. This pattern may be attributed to managerial practices and working conditions, such as increased workload and limited ability to claim professional rights. This interpretation is supported by the high mean scores for items such as “Being exposed to an unmanageable workload” and “Pressure not to claim something to which you are entitled.” These findings underscore the critical role of effective leadership in promoting healthy working conditions.

Person-related bullying emerged as the second most frequent form, involving negative acts directed at the individual on a personal level. This may be linked to organizational cultural factors, particularly communication patterns among nurses. Supporting this interpretation, the highest mean score within this subscale was observed for the item “Repeated reminders of your errors or mistakes.” This highlights the need to foster a positive organizational culture characterized by a just culture framework and effective professional communication. Finally, physically intimidating bullying was identified as the least frequent form, involving overtly aggressive or threatening behaviors. Within this subscale, the highest mean score was associated with the item “Being shouted at or being the target of spontaneous anger.” Although less prevalent, these behaviors remain concerning and indicate the importance of reinforcing professional conduct standards. Accordingly, healthcare organizations should prioritize the promotion of respectful workplace behaviors and the prevention of all forms of workplace bullying.

The observed inverse association between authentic leadership and workplace bullying suggests that stronger perceptions of authentic leadership may be linked to a less bullying-prone work environment. This negative correlation was evidenced in previous research conducted in various countries [19,21–24]. Notably, the observed correlations extended across all dimensions of workplace bullying—work-related, person-related, and physically intimidating—suggesting that authentic leadership exerts a broad influence on all aspects of the workplace bullying. Similarly, all subcomponents of authentic leadership demonstrated moderate negative correlations with workplace bullying, indicating that its multidimensional nature operates in an integrated rather than isolated manner.

The current study underscores the significant association between authentic leadership and workplace bullying in hospital settings. This relationship can be interpreted in light of the foundational principles of authentic leadership, which emphasize the development of positive psychological capacities and the promotion of an ethical and supportive work climate [26], which may discourage the emergence or persistence of bullying behaviors. Such positive leadership approach fosters trust, strengthens collegial relationships, and contributes to reducing stressful work environments, thereby decreasing the likelihood of bullying behaviors [8,11,13,42]. In contrast, prior research has shown that autocratic, unsupportive, and disengaged leadership styles contribute to power imbalances and an increased incidence of workplace bullying [13]. Collectively, these findings contribute to the growing body of evidence supporting the positive impact of authentic leadership on improving the work environment and promoting professional behaviors in healthcare settings.

In relation to the predictors of workplace bullying in Jordanian hospitals, the present study identified hospital type, working unit, and the transparency dimension of authentic leadership as significant predictors. These results differ from those reported by Obeidat et al. [9] who identified age and sex as significant predictors of workplace bullying. The discrepancy between results may reflect contextual differences across healthcare institutions.

The lower level of workplace bullying reported by nurses in private hospitals may reflect differences in organizational structures, managerial practices, workplace policies, or organizational culture across hospital sectors. Differences in institutional priorities and approaches to staff management may also influence how workplace behaviors are experienced and addressed. Similarly, the higher level of workplace bullying reported among nurses working in emergency departments may reflect differences in the clinical and organizational context of emergency care. The fast-paced and unpredictable nature of emergency departments, together with time pressure, high patient turnover, complex team interactions, and frequent exposure to emotionally demanding situations, may create conditions in which interpersonal tensions are more likely to arise. However, these organizational and contextual factors were not directly assessed in the present study; therefore, they should be regarded as possible explanations rather than established mechanisms underlying the observed differences. Future research should examine organizational and unit-level factors to better understand the mechanisms contributing to workplace bullying across hospital sectors and clinical settings in Jordan.

The study further demonstrated that nurses who perceived their managers as exhibiting higher levels of transparency were less likely to report workplace bullying. This finding is consistent with prior national and international studies identifying authentic leadership as a significant predictor of workplace bullying [19,21,22,24]. Importantly, the present study extends the existing literature by identifying transparency as a significant dimension of authentic leadership associated with workplace bullying. This finding may be particularly relevant because transparent leadership emphasizes openness, clarity, honesty, and consistency in communication and decision-making. In the context of nursing, such behaviors may strengthen trust and reduce ambiguity regarding expectations, managerial decisions, and interpersonal interactions, thereby contributing to a work environment less conducive to conflict and negative interpersonal behaviors.

One possible explanation is that transparent communication provides nurses with greater clarity regarding managerial decisions and how workplace concerns are addressed, which may reduce misunderstandings and perceptions of unfairness that can contribute to interpersonal tension. Transparency may also foster an environment in which nurses feel more comfortable expressing disagreement, asking questions, and raising concerns without fear of negative interpersonal consequences. Such openness may facilitate the early identification and constructive management of interpersonal tensions before they develop into persistent patterns of negative workplace behavior. However, these mechanisms were not directly examined in the present study and should therefore be considered plausible explanations rather than established pathways. Future research, particularly longitudinal and mediation studies, is needed to clarify the mechanisms through which leadership transparency may be associated with workplace bullying.

## Implications and recommendations

The present study identified a high prevalence and elevated levels of workplace bullying, highlighting an important concern within Jordanian healthcare settings. These findings underscore the need for healthcare policymakers and hospital administrators to adopt structured, evidence-informed approaches to prevent and address workplace bullying. The observed inverse association between authentic leadership and workplace bullying further suggests that leadership practices may represent an important organizational target for intervention. Accordingly, hospitals could consider implementing structured authentic leadership workshops for nurse managers. Given that transparency emerged as a significant predictor of workplace bullying in the present study, leadership development should specifically strengthen managers’ ability to communicate decisions and expectations clearly, provide consistent information, encourage nurses to voice concerns, and respond openly to workplace problems. Individualized manager coaching and periodic 360-degree feedback could complement these workshops by helping nurse managers identify potential gaps between their intended leadership behaviors and nurses’ perceptions of those behaviors.

Organizational strategies should also include formal anti-bullying policies, designated anti-bullying committees or responsible personnel, and confidential and accessible reporting systems with clearly defined procedures for documenting, investigating, and responding to reported incidents. Regular workplace-climate assessments could be used to identify units with higher levels of bullying and to monitor changes following intervention. Workplace-bullying prevention strategies may also need to be tailored to the organizational and clinical contexts rather than applied uniformly across all units. Although these strategies may provide practical avenues for strengthening leadership and addressing workplace bullying, their effectiveness should be evaluated through longitudinal and intervention studies. Such research could determine whether improvements in authentic leadership, particularly transparency, are followed by reductions in workplace bullying and improvements in nurses’ work-related outcomes.

## Limitations

Despite the valuable contributions of this study, several limitations should be noted. First, the cross-sectional design restricts the ability to establish temporal or causal relationships between authentic leadership and workplace bullying. Second, the use of convenience sampling may have introduced selection bias, as participating nurses may differ from those who were not included in the study. Third, the study was conducted exclusively in private and university-affiliated hospitals in Amman and did not include public hospitals or healthcare institutions in other geographical regions of Jordan. Therefore, the generalizability of the findings to nurses working in other hospital sectors and regions may be limited, particularly where organizational structures, leadership practices, and working conditions differ. Additionally, the use of self-reported measures may have introduced response or social-desirability bias, particularly given the sensitive nature of workplace bullying. Future studies should include public, private, and university-affiliated hospitals across different regions of Jordan. Longitudinal, qualitative, or mixed-methods approaches may also provide deeper insight into the mechanisms underlying the relationship between authentic leadership and workplace bullying.

## Conclusion

The current study contributes to the growing body of literature emphasizing the critical role of authentic leadership in shaping workplace dynamics within healthcare settings. The results demonstrate a significant inverse association between authentic leadership and workplace bullying, indicating that higher levels of perceived authentic leadership were associated with lower levels of workplace bullying among nurses. Furthermore, the identification of hospital type, working unit, and the transparency dimension of authentic leadership as significant predictors of workplace bullying underscores the multifactorial nature of this phenomenon.

The study further suggests that interventions targeting only individual perpetrators or victims may be insufficient for effectively addressing workplace bullying. Instead, healthcare organizations should adopt comprehensive and multilevel strategies that address structural workplace conditions, promote authentic and transparent leadership practices, improve staffing and workload management, and foster positive organizational cultures. These findings highlight the potential value of addressing both leadership and organizational factors in workplace-bullying prevention and in promoting healthier nursing work environments.

## Supporting information

S1 Checklist Inclusivity in global research. (DOCX)
